# Realizing the right to health in Brazil’s Unified Health System through the lens of breast and cervical cancer

**DOI:** 10.1186/s12939-019-0938-x

**Published:** 2019-06-03

**Authors:** Fabiana da Mota Almeida Peroni, Magnus Lindelow, David Oliveira De Souza, Mirja Sjoblom

**Affiliations:** 1 Universidade Estadual de Campinas, Campinas-SP, Brazil; 2 Health Nutrition and Population Global Practice, The World Bank, Washington, DC, USA; 3 Global Health Policy Unit, The University of Edinburgh, Edinburgh, UK

**Keywords:** Right to health, Brazil, Bahia, Cancer care, Cervical cancer, Breast cancer, Health system, Brazil’s Unified Health System (Sistema Único de Saúde, SUS), Equity

## Abstract

**Background:**

Health is recognized as a fundamental right in Brazil’s constitution. In the absence of a clearly defined benefit packages of healthcare services that are financed under the Unified Health System (Sistema Único de Saúde, SUS), courts have become important in adjudicating coverage decisions. Empirical assessments of equity and the right to health tend to focus on simple measures of access. However, these empirical perspectives belie the significant inequalities and rights violations that arise in the case of more complex health needs such as cancer. To shed light on these issues, this paper focuses on the care pathways for breast and cervical cancer and explores access and quality issues that arise at different points along the care pathway with implications for the realization of the right to health in Brazil.

**Method:**

A mixed method approach is used. The analysis is primarily based on a quantitative analysis of national representative administrative data principally from the cervical and breast cancer information systems and the hospital cancer registry. To gain more insights into the organization of cancer care, qualitative data was collected from the state of Bahia, through document analysis, direct observation, roundtable discussions with health workers (HWs), and structured interviews with health care administrators.

**Results:**

The paper reveals that the volume of completed screening exams is well below the estimated need, and a tendency toward lower breast cancer screening rates in poorer states and for women in the lowest income brackets. Only 26% of breast cancer cases and 29% of cervical cancer cases are diagnosed at an early stage (stage 0 or I), thereby reducing the survival prospects of patients. Waiting times between confirmed diagnosis and treatment are long, despite new legislation that guarantees a maximum of 60 days. The waiting times are significantly longer for patients that follow the recommended patient pathways, and who are diagnosed outside the hospital.

**Conclusion:**

The study reveals that there are large variations between states and patients, where the poorest states and patients fare worse on key indicators. More broadly, the paper shows the importance of collecting data both on patient characteristics and health system performance and carry out detailed health system analysis for exposing, empirically, rights violations and for identifying how they can be addressed.

**Electronic supplementary material:**

The online version of this article (10.1186/s12939-019-0938-x) contains supplementary material, which is available to authorized users.

## Background

With the prominence given to the achievement of Universal Health Coverage (UHC) in the Sustainable Development Goals framework, the right to health and its legal enforcement are increasingly relevant [1]. As stated by the World Health Organization (WHO) UHC is defined as “all people receiving quality health services that meet their needs without being exposed to financial hardship in paying for the services” [2]. Thus, UHC is inclusive of effective coverage, i.e., access to quality health services (as opposed to insurance coverage) and protection against financial risk. UHC has been called a “practical expression of the right to health” [3]. As countries progress towards UHC, policy-makers face difficult choices related to which services to expand first and to whom. Litigation over rights to goods and services will certainly play a role in how UHC is implemented, particularly in countries where the right to health is encoded in the national laws [1].

The right to health as set forth in the WHO Constitution [4] is defined as “the highest attainable standard of health”. Braveman and Gruskin [5] argue that the highest attainable standard of health can be understood to reflect the standard of health enjoyed by socially advantaged groups within a society, since these could be possible for everyone living in that society. This means that equity in health helps to operationalize the concept of the right to health. WHO defines health equity as “the absence of avoidable, unfair, or remediable differences among groups of people, whether those groups are defined socially, economically, demographically or geographically or by other means of stratification” [6].

In the case of Brazil, health is recognized as a fundamental right in Brazil’s 1988 constitution and the universal right to health is a founding principle of the Unified Health System (Sistema Único de Saúde [SUS]). Guided by the constitutional principles of universality, comprehensiveness/continuity of care and equality of access to health care, guaranteeing the right to health is a shared responsibility of Brazil’s three governmental spheres (federal, state and municipal) [7].

On the one hand, the constitutional right to health can have positive effect on equity in access to healthcare if individuals who are denied access to specific medicines or technologies (due to e.g. health system delivery shortfalls or delays in inclusion of medications into SUS) turn to the courts to gain access and thereby uphold the principles of universality encoded in SUS. On the other hand, judicialization may increase inequity, if litigation leads to the careless use of medicines or services, or if individuals with better socio-economic standing who may last longer in litigation get priority access to medicines and health services at the expense of poorer populations. In such instances, litigation may violate the principles of SUS [7] and ultimately the power to make public policy is moved from policy-makers to the courts [8].

The inception of SUS led to profound changes in the healthcare system in Brazil with significant achievements, including the rapid expansion of primary care, the integration of several independent systems of financing and service provision into a single publicly funded system, and increases in government spending on health (with particular focus on basic care) [9]. As a result, large improvements in health service coverage and health outcomes have been observed [10, 11]. However, disparities in access to healthcare and health outcomes persist, with a higher level of utilization among high-income groups and unmet healthcare needs in populations living in poorer northern regions [9]. Largely, health inequalities mirror income inequalities in the country [12].^1^

To operationalize the right to health in SUS, the government increased the health facility network and maintained the legal provision that anyone should have access to an open-ended benefit package free-of-charge under the SUS [9]. Effectively, this implies that the government supply any medication judicially ruled, even if that conflicts with established public policy such as clinical protocols and therapeutic guidelines [13]. The Judiciary has even made recent calls to have clear and objective parameters regarding which medications are covered by SUS [ibid].

In the absence of clear standards, consensus or criteria for which services and technologies should be financed within SUS, the courts have become important in adjudicating coverage decisions. Right-to-health litigation has increased exponentially. While data at the national level is not available, data from 7 out of 26 states showed that federal spending on court-ordered health services increased 40 times since 2005, reaching an estimated USD $550 million in 2010 [14]. However, these numbers are not inclusive of most of the costs that occur at the state and municipal levels. Most cases are individual ones (only around 3% are collective) that focus on access to health care services (mostly drugs), and the success rate for these litigants is high [15]. A growing body of evidence shows that judicialization accentuates inequalities in the health system. Most of the court orders are in states with the highest Human Development Index [15], and evidence from the municipality of São Paulo show that litigants tend to originate from the neighborhoods with the lowest levels of exclusion or social vulnerability [16].^2^

The legal system may, however, be a slow and costly mechanism for making decisions about health care coverage. Whether the right to health is delivered is determined in the more complex world of how services are organized and delivered, where advances in the right to health could arguably be made through improvements in systems and management in some cases without additional resources. To operationalize the right to health, it is therefore critical to understand and ensure if and how the health system is fair [8], and if and how it provides equal opportunity for all [17].

Empirical assessments of equity and the right to health tend to emphasize simple measures of access or utilization of services. Similarly, much of health-related litigation focuses on access to specific drugs or procedures. However, these empirical perspectives and manifestations of a rights-based approach belie the significant inequalities and rights violations that arise in the case of more complex health needs such as cancer. To shed light on these issues, the paper focuses on the care pathways for breast and cervical cancer and explores access and quality issues that arise at different points of the care pathway and at different levels of the health system with implications for the realization of the right to health in Brazil.

Cancer is a growing health challenge in Brazil and the annual number of deaths from cancer increased from fewer than 100,000 in 1990 to around 200,000 in 2013, representing 17% of all deaths [18]. Estimates of incidence rate for 2018 show that with the exception of non-melanoma skin cancer, breast (29.5%) and cervical cancer (8.1%), together with colorectal cancer (9.4%), are the most common cancers for women [19]. With the large anticipated increase in the number of elderly in Brazil (the share of the population 60 years and older is projected to increase from 10.2% in 2010 to 29.3% in 2050 [20]), the number of cancer cases is likely to increase in the future. Furthermore, lawsuits related to cancer care represent a significant part of litigation and are a growing burden on the public purse [21–23]. It is therefore important to understand the performance of SUS related to cancer care.

Previous literature focuses on measuring delays in cancer care and casual factors related to delays, primarily for breast cancer [24–28]. Studies in various states show that a large proportion of women with breast cancer are diagnosed in late stages (II-IV) [24, 25, 27]. There is only one national study to date that shows that 60% of cancer patients received their diagnosis in a late stage (III or IV) and only 15.9% of patients received treatment within 30 days, with an average wait time for radiotherapy of 113.4 days in 2010 [28]. Factors related to late diagnosis include lack of patient awareness of cancer symptoms, difficulty in obtaining access to diagnostics procedures and specialist care [24, 26, 28] and deficiencies in primary care [28]. Simon et al. (2009) find that patients diagnosed with breast cancer in public institutions and in the poorer north of the country were diagnosed at later stages than those diagnosed in the private sector and in the wealthier south of Brazil [27].

The paper contributes to this field of research by providing new analysis of administrative data in a number of areas including waiting times, stage of diagnosis, and measurements of productivity of cancer care by state and region along the patient care pathway. In addition to updating and expanding information in the only available national study from 2011 [28], it includes additional information on prevention, screening and diagnosis, various productivity measures, and analysis along socio-economic dimensions where data permits. Based on qualitative data from one state (Bahia) it sheds light on the underlying factors behind the patterns observed in the quantitative data. More broadly, the paper highlights the importance of health system analysis for exposing, empirically, rights violations and ultimately the distribution of health and ill health within and across societies.

## Methods

The paper focuses on the patient care pathways for breast and cervical cancer and explores access and quality issues that arise at different points of the care pathway and at different levels of the health system with implications for the realization of the right to health in Brazil.

The paper uses a mixed method with triangulation of national quantitative data and qualitative information from one state (Bahia). The study is primarily based on analysis of administrative data collected at both national and state levels through cancer information systems (SISCOLO and SISMAMA) and provider-based record systems.

SISCOLO and SISMAMA are national information systems for cervical and breast cancer and capture information of results from both diagnostic and screening exams (mammography and Pap smear) and pathological exams of the breast and uterus (biopsy and surgical specimen). These systems contain data from public providers, private providers contracted by the SUS, states and municipalities. They are comprehensive and reliable for publicly financed health care in Brazil. However, SISCOLO and SISMAMA do not cover an estimated 24.4% of the population with private health insurance that access health care through private providers and may therefore not adequately represent the more affluent states in the south (e.g. Federal District, Sao Paolo and Rio de Janeiro) where private health insurance is high (above 20%) [29].

Official population-based parameters were used to estimate health service needs, with adjustments made to account for the share of population covered by private health providers. The parameters to estimate those needs were based on official guidelines from the Ministry of Health (MOH), protocols for screening and treatment and guidelines from the Brazilian National Cancer Institute (INCA). Furthermore, national data from the Outpatient Information System (SIA) provided data on procedures outside the hospital, while the Hospital Cancer Registry (HCR) was used to capture advanced diagnostics procedures, surgery and other treatment procedures administered at the hospital level. Data on infrastructure, health professionals and other elements of the health system was obtained from the Registry of health facilities (Cadastro Nacional de Estabelecimentos de Saúde [CNES]) to analyze resources available for cancer care. Data from the national household survey (Pesquisa National por Amostra Domicililos [PNAD]) was also used in the study. Data from HCR and CNES covers all health facilities in the country, including privately owned facilities, and is national representative. Data related to the delivery of cervical and breast cancer care was collected from these various national administrative systems and analyzed to characterize the realization of the right to health for these two cancers in Brazil. A summary description of national data sources is included in Table 1.

**Table 1:** Summary description of national data sources

	Description	Comments
National information systems for cervical and breast cancer (SISCOLO/SISMAMA)	Information systems for cervical and breast cancer until 2012, when they started to be integrated in new cancer information system (SISCAN). The information system is fed by public providers, private providers contracted by the SUS, states and municipalities. The system captures data on the results of mammography, Pap smear exams and pathological exams of the breast and uterus (biopsy and surgical specimen).	The system suffers from delays in data entry and incomplete data for some variables (with variation across states).
Hospital Cancer Registry (HCR)	Fed by hospitals and provides the basis for financial transfers to providers. Captures advanced diagnostics procedures, surgery and other treatment procedures that are administered at the hospital level.	Given the link with financial transfers, data tends to be more complete. Incentives for over-reporting exist but are mitigated by control and auditing systems.
Outpatient Information System (SIA)	Covers all procedures provided outside hospitals. Data entered by providers. Provides the basis for financial transfers to providers. Includes data on all outpatient procedures.	Given the link with financial transfers, data tends to be more complete. Incentives for over-reporting exist but are mitigated by control and auditing systems.
National Household Sample Survey (PNAD)	Is the national household sample survey conducted by Brazilian Institute of Geography and Statistics since 1981. The study draws on national representative data from the health section.	2008 was the last time the health section of PNAD was conducted. New data, including the health module, was collected in 2018 but is not yet available.
Registry of health facilities (CNES)	The system provides infrastructure information, type of care provided, specialized services existing beds and the number of health professionals in health facilities.	Although the CNES provides information on infrastructure and HR, it does not contain data on performance.
Parameters of needs	The parameters to estimate the needs in the study have been based on official guidelines from MOH described in different ordinances (1101/2002 and others), protocols for screening and treatment and guidelines from INCA.	MOH has calculated those parameters based on scientific literature and previous years data.

Source: Authors

To compliment the quantitative analysis and gain more insights into the organization of cancer care, qualitative data was collected in the state of Bahia. Bahia is an important state in northeast Brazil, which has a population of 14.3 million people, with about 2.6 million (1.1 million) citizens living below the moderate (extreme) poverty line. Bahia was chosen after consultations with the MOH and INCA because it represents an average performing state in cancer care. Given the large size of the state it was necessary to narrow the geographical scope of the qualitative study to one of Bahia’s nine health macro regions – the east macro region. This region was selected because it represents diversity both in terms of geography and economy and it is also where the state capital is located with important cancer facilities that receive referrals from other municipalities in the state. The east macro region has four health regions (Camaçari, Cruz das almas, Salvador e Santo and Antônio de Jesus) and 48 municipalities and is home to 4.4 million people.

The study in Bahia was based on document analysis, data from SUS information system, roundtable discussions with health workers, structured interviews and focus group discussions with staff working in health care administration in the state, and semi-structured interviews and direct observations in health facilities. To better understand the organization of cancer care and the situation facing HWs, and identify challenges perceived by the HWs, roundtable discussions with HWs were conducted in the four health regions of focus.

Three roundtable discussions were held in each health region with a total of 116 participants. Data collection took place from July to September 2014. At least one HW from each of the four areas (primary care, specialized care, regulation and control and information systems) was selected by the local manager to get diverse perspectives. The questionnaires used during the roundtable discussions were designed in collaboration with managers and technical staff from the MOH, INCA and the State Heatlh Secretariat of Bahia (SESAB). The discussions were led by a Brazilian researcher and focused on the following topics: policy context at the federal and state level, the characterization of the network of cancer care for the two cancers and system performance measures. A summary description of qualitative data collected in the state of Bahia is included Additional file 1: Table S1.

An innovative feature of the study was that realistic patient cases were discussed, and specific questionnaires were used to identify patient pathways and obstacles to accessing care during roundtable discussions. To identify weaknesses in system design and identify patient pathways, structured interviews (based on questionnaires focused on access, regulation, contracting and primary as well as specialized care) and focus-group discussions were undertaken with 17 participants from SESAB, the municipality of Salvador and the State Center for Oncology. Direct observations and semi-structured interviews at the facility level were conducted in three facilities to identify how to get access to screening tests, specialized appointments, etc. All interviews and roundtable discussions were recorded, transcribed and analyzed. Furthermore, administrative data was examined, and a review of policies and programs was conducted. Equity in access to care was explored and considered throughout the analysis of the data. The preliminary results of the study were also presented and discussed with policy makers in Bahia and at the national level to confirm the results and strengthen the rigor of the analysis. The study was approved by the Research Ethics Committee of SESAB under No. 091582/2014, and participants agreed to be part of the study by signing the Informed Consent Form.

## Results

Drawing on the Organization for Economic Co-operation and Development (OECD)’s framework for elements of good cancer care [30] this section is focused on the patient care pathway for breast and cervical cancer. To set the stage, the first section discusses the trends in breast and cervical cancer in Brazil. Subsequent sections focus on effective prevention, early detection and screening, accurate diagnosis and staging and prompt access to treatment. These represent elements of good cancer care. The final section discusses resources available for cancer care in Brazil.

### Trends in breast and cervical cancer

**Fig. 1:**
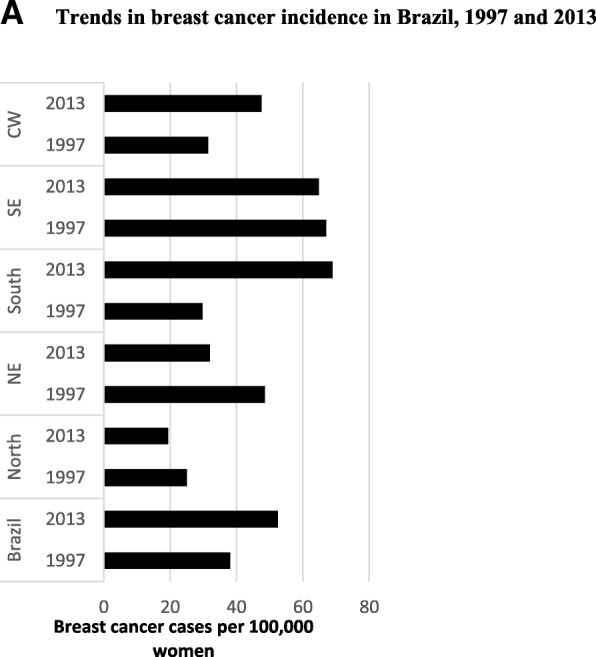
Cancer incidence in Brazil, 1997 and 2013

**Fig. 2:**
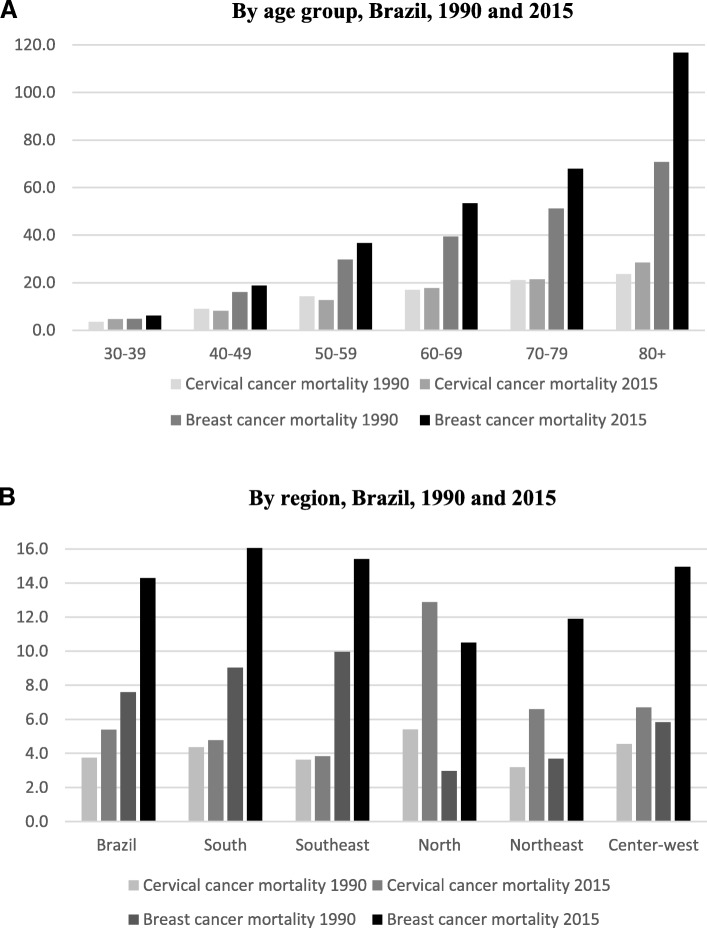
Trends in breast and cervical cancer mortality rates/100,000 women

The incidence of breast cancer in Brazil increased between 1997 and 2013, while the incidence of cervical cancer declined (Fig. 1). There are important differences in trends across regions; the increase in the breast cancer incidence was highest in the south and central west. For cervical cancer, the incidence decreased in all regions except the south, where incidence has been stable. Per Fig. 2 mortality rate for breast cancer increased from 7.5 to 14.3 deaths per 100,000 women between 1990 and 2015. Similarly, although with less magnitude, the mortality rate for cervical cancer also increased from 3.8 to 5.4 deaths per 100,000 women for the same period. For breast cancer, the mortality rates have increased in all age groups (except between ages 10–14) with large increases for women over 60 years old, and particularly for those over 80 years old. Mortality rates for cervical cancer decreased for women between 40 and 59 years old as well as for those age 10–14, however, it increased again for women over 80 years old. There are also notable regional differences in mortality rates. For cervical cancer mortality rates have increased rapidly in the poorest northern region. For breast cancer mortality there are deteriorations in all states within the second poorest region (central west) and the poorest region (northeast), with the highest increases. Relative cancer survival rates are a good indicator of the performance of the health system in cancer care. However, because there is no population-based cancer registry in Brazil, survival rates are not available.^3^ The absence of individual data makes it impossible to determine if these two cancers disproportionately affect certain population groups or individuals.

### Effective prevention

According to global estimates from the WHO, about 30–50% of cancer cases are preventable [31]. This highlights the importance of prevention in effective cancer control. For all cancers, important risk factors for cancer are the prevalence of overweight, tobacco smoking and alcohol consumption.

With regards to specific strategies to prevent cervical cancer and breast cancer, SUS has introduced Human papillomavirus (HPV) vaccination to prevent cervical cancer, and genetic testing to better screen for breast cancer. The Brazilian HPV vaccination protocol is similar to those seen in other countries with three doses of the vaccine offered over a period of 5 years. The national coverage rate for the second vaccine dose in 2014 was 59.3% of the target population in the country [32]. Genetic testing that assesses the risk of breast cancer (BRCA1 and BRCA2) is currently only offered in university hospitals or highly specialized centers to help identify women with a family history of cancer who would benefit from early screening. Population-based awareness campaigns focused on breast, cervical and lung cancer have been administered by MOH but on an irregular basis.

### Early detection and screening

If cancer can be diagnosed prior to the onset of symptoms, there are more treatment options and better survival prospects. Thus, screening is targeted at an asymptomatic population to detect suggestive or precursor lesions of cancer that can be referred for diagnostic investigation. In Brazil, SUS offers population screening for breast and cervical cancers free of charge. For cervical cancer, Pap smear tests should be available to women between 24 and 64 years old. The national guidelines recommend annual tests, but when there are two consecutive negative tests, a woman can delay subsequent testing for 3 years. To detect breast cancer, annual clinical breast exams are recommended for women over 40 years of age and mammography screening is recommended for women between 50 and 69 years of age every 3 years. These guidelines are in line with most OECD countries, although the age bracket used to define the target group varies between countries [30].

To obtain a proxy for coverage rates of mammography screening, the volume of mammography screening exams completed is put in relation to population-based estimates of individuals in need of mammography exams. At the national level 65% of the need for screening is met. There are also large variations within the country, where relatively wealthy states (Sao Paolo [97%], Santa Catarina [94%] and Parana [88%]) have high levels of coverage, in contrast to the worst performing states that only cover a small percent of the population, e.g. Amapa (0%) and Para (15%).

- Interestingly, administrative data shows that almost half (46%) of all mammogram screenings performed in SUS are for women outside the target population. Qualitative data from Bahia confirmed this finding and shows that the state has a flexible targeting strategy: *“The MOH recommended age for screening of breast cancer is 50 to 69 years; however, we see an increasing number of cases detected below this age group. The municipality of Salvador, and several other municipalities, decided to be more flexible and execute mammography screening after age 35. We also changed the upper age limit, because life expectancy is increasing for women, to include women between 70 and 80 years old.”* This is an example of how health professionals do an implicit prioritization of women outside the target population as per stipulated in the national policy and in WHO recommendations [33].

**Fig. 3:**
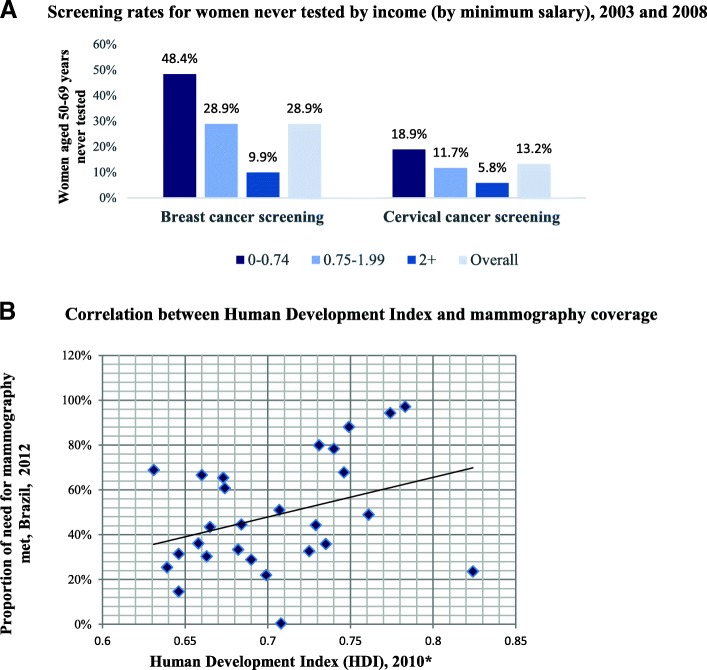
Screening rates for women never tested by income (by minimum salary), 2003 and 2008

For cervical cancer, the volume of completed screening exams is also well below the estimated need. At the national level, 54% of the estimated need of Pap smear exams is met, with significant variation across states. However, in most states the number of Pap smear cytology samples collected exceeds the number of tests completed, in some cases by a large margin. This is likely related to issues with the quality of Pap smear exams as described in detail below. Coverage of breast and cervical cancer screening is lower in the poorest socioeconomic groups. In national representative survey-based data from the PNAD (Fig. 3a) three socioeconomic groups are defined: the poorest (III) living on 0–0.74 times the minimum salary; medium poor (II) living on 0.75–1.99 times the minimum salary; and (I) 2 or more times the minimum salary. Figure 3a shows that almost half of the women in the lowest socioeconomic group (48.4%, Confidence Interval [CI] [47.1, 49.6]) had never received breast cancer screening, compared to 28.9% (CI [28.0, 29.8]) in the next to highest socio-economic group (II) and 9.9% (CI [9.1, 10.7] in the highest socio-economic group (I). The difference between access levels of socioeconomic groups is less in cervical cancer screening. About 18.9% (CI [18.3–19.5] of women in the poorest group had never received a Pap smear, compared to 11.7% (CI [11.2–12.2] in group II and 5.8% in group I (CI [5.3–6.2]). This finding is also confirmed by state level data that shows a positive correlation between state level mammography coverage and the state’s human development index (HDI) (Fig. 3b). This suggests that states that are better off economically have higher coverage rates of breast cancer screening and that groups with low socioeconomic status are systematically disadvantaged.

### Accurate diagnosis and staging

The optimal time to diagnose breast and cervical cancer is as soon as the cancer is detectable by screening. When the cancer has already become symptomatic the therapeutic options are more limited. Early diagnosis of cancer requires careful clinical evaluation and laboratory tests, especially biopsies, to confirm diagnosis and determine the stage of the tumor in order to determine treatment options.

**Fig. 4:**
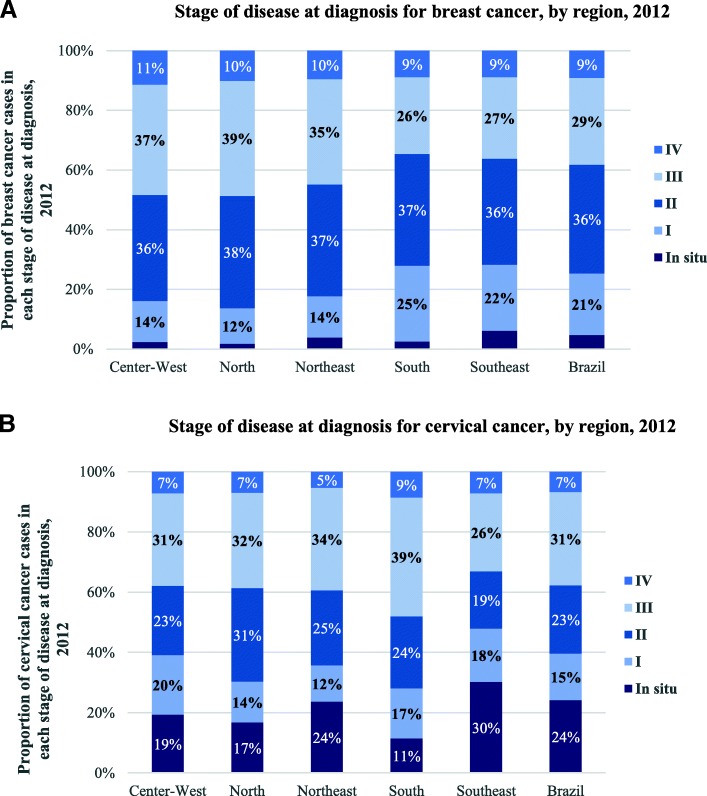
Stage of disease at diagnosis for breast cancer, by region, 2012

Using national level HCR data, stage of diagnosis for breast and cervical cancer was estimated by state and region (Fig. 4a and b). Very few cases of breast (26%) and cervical (39%) cancer are diagnosed at an early stage (stage 0 or I), thereby reducing survival prospects of patients. The stage of diagnosis also varies substantially between different states, particularly for cervical cancer. The relatively wealthy state of São Paulo provides confirmed diagnosis to 60% of women with cervical cancer in stage 1, while the equivalent number for some of the poorest states in the country (Alagoas, Acre and Roraima) is 10%. Significant variations exist even within the same region of Brazil. In the south, Rio Grande do Sul and Santa Catarina diagnose 20% of cervical cancers in stage 1, while Paraná performs much better with 40% of its cervical cancers diagnosed at the same time. Variations exist for breast cancer as well, although they are smaller than for cervical cancer. Because there is no national cancer registry in Brazil, it is not possible to determine if certain population groups face additional barriers for early diagnosis. But since poorer citizens are disadvantaged as far as screening for breast and cervical cancer is concerned, it is likely that such inequities persist for the timing of diagnosis since screening programs are meant to detect individuals with cancer risk.

Several health system bottlenecks were identified that can contribute to late stage diagnosis including:

- *Low access to more sensitive diagnostics procedures:* An analysis of administrative data on the volume of biopsy procedures relative to population-based estimates of needs shows that only 35% of biopsy needs are met in Brazil, with large variations between states from 78% in Sao Paolo to 1% in Amazonas. Coverage for Fine Needle Aspiration (FNA) for diagnosis of breast cancer is similarly low at 27%.
- *No effective prioritization of patients with more advanced cancer:* 2014 data from SISMAMA shows that the time interval between the request for a specialist appointment and the completion of the exam is almost the same for patients with a mammogram that shows clinical symptoms and patients that are asymptomatic. Thus, the system does not give priority to patients that have more advance stages of cancer.
- *Challenges regarding the quality of diagnostic exams (both the collection process and analysis of results):* The number of Pap smear cytology samples collected exceeds the number of tests completed, in some cases by a large margin. At the national level, collected samples represent 100% of the need, but only 54% of these exams are completed. Thus, a large share of collected samples are never completed and may therefore need to be repeated. This may be a result of how challenging it is to obtain quality Pap smear exams or tests being lost between the unit that collects the sample (often primary care) and the entity that carries out the diagnostics. This is also confirmed by the qualitative data. Focus group discussions with HWs in Bahia highlighted that poor clinical skills among HWs in primary care affect the quality of diagnostics, particularly for Pap smear exams. One HW explained: “*With regard to cervical cancer there are several issues. The first issue concerns HWs’ knowledge in carrying out Pap smear tests. We observed that there were many unsatisfactory samples and even after receiving training we haven’t achieved the expected results*.” There also seem to be issues with task-shifting that may affect the quality of diagnosis: *“It is important to point out that currently most Pap smear exams are performed by nurses in primary care, because the doctors who are trained to do this test have not performed the exam. Many of the nurses are recent graduates that are left alone to try to figure out how to carry out these exams.”* Of the eleven patient cases collected in the qualitative material from Bahia, four women had to repeat the test because of the poor quality of exams. Even if this small sample is not representative, the extent of repeat exams raises concerns about the quality of diagnostics procedures.

### Prompt access to treatment

**Fig. 5:**
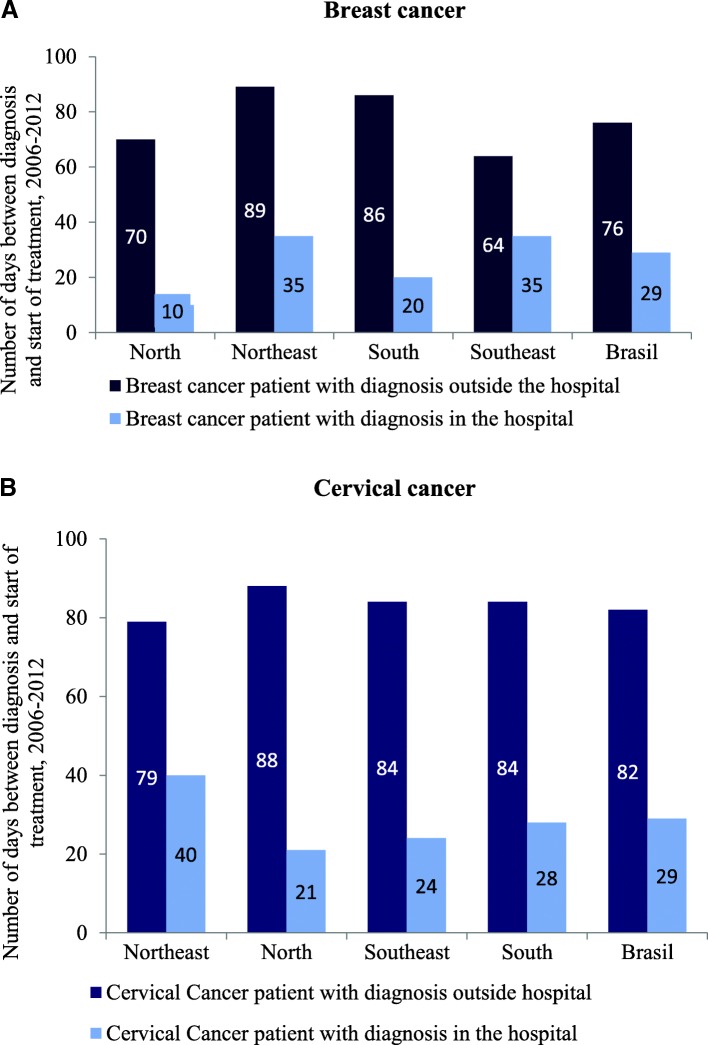
Days between diagnosis and treatment by location of diagnosis, by region, 2006–12 (averages)

A waiting time legislation was introduced in 2014 that guarantees treatment within 60 days of a confirmed diagnosis (based on the diagnosis being recorded in the medical record).^4^ This is a long time compared to many OECD countries – e.g. Chile (30 days for breast cancer and 20 days for cervical cancer), Czech Republic (4 weeks) and England (31 days) [23]. Despite the waiting time legislation, there is little systematic data on waiting times in Brazil. Available data from HCR focuses on the time between confirmed diagnosis and treatment and suggests that waiting times are long and vary significantly depending on the patient pathway. There are large differences in waiting times between patients that are diagnosed through the recommended patient pathway, i.e. diagnosis outside the hospital and referral to treatment in the hospital, relative to patients that proceed directly to the hospital for diagnosis and treatment (76 vs. 29 days for breast cancer and 82 vs. 29 days for cervical cancer) (Fig. 5a and b). For patients that are diagnosed outside the hospital, only eight states meet the waiting time guarantee for cervical cancer patients (Acre, Alagoas, Ceara, Goias, Mato Grosso, Mato Grosso do Sul, Piaui and Rio Grande do Norte) and only seven states meet it for breast cancer patients (Ceara, the Federal District, Espirito Santo, Goias, Piaui, Rio Grande do Norte and Roraima). All states (except Sergipe for cervical cancer) meet the waiting time guarantee of 60 days between diagnosis and treatment when patients go directly to the hospital. Thus, unless the patient turns to the hospital for diagnosis, the waiting time guarantee is not met, and this may explain why many patients skip primary care and go directly to high-complexity units.

For both breast and cervical cancers, surgery is the most common treatment when the cancer is detected early, while radiotherapy and chemotherapy are used more often for cancers diagnosed at later stages. Consistent with the long waiting times and the advance stage at which many women receive confirmed diagnoses, 2014 data from the HCR shows that in most municipalities, a larger share of women start with chemotherapy (82.2%) compared to surgery (50.6%). Previous research shows that SUS users of breast cancer treatment services travel 67 km to access treatment and 75% of displacements between municipalities were closer than 151 km away (Oliveira et al., 2011). Since treatments with e.g. radiotherapy require frequent visits and there are long distances for patients to travel, patients with cancer may face logistical, financial or other challenges related to their travel to the treatment unit. This was also confirmed in qualitative data that shows that the costs for transport, accommodation, food and the opportunity costs for not working are important barriers to access cancer care and for the ability of patients to adhere to treatment regimes.

### Resources available for cancer care

At first glance, cancer care in Brazil is relatively well resourced from an international perspective. Although Brazil has fewer oncologists per million people (24) than Sweden (61) and the United States (36), it is better resourced in terms of the number of oncologists at a national level than many OECD countries such as France (11) and Turkey (4) (OECD, 2013). Regarding technology (Computerized Tomography [CT] scans and Magnetic Resonance Imaging [MRI] units per million people), Brazil compares unfavorably with the United States and South Korea but appears to have more technology for effective cancer care than many other middle- and high-income countries such as the United Kingdom and Mexico [30].

**Fig. 6:**
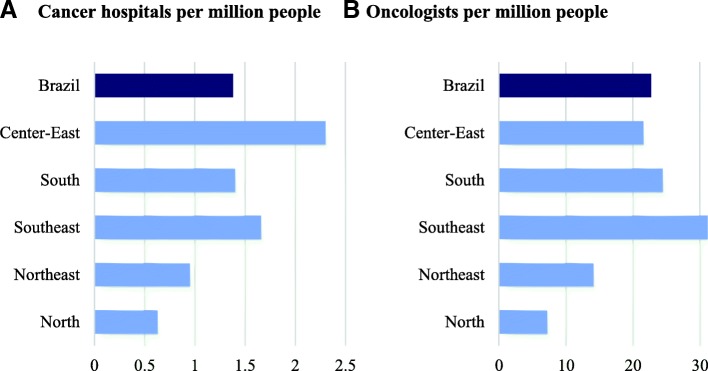
Resources for cancer care, by region, 2013

- However, more detailed analysis reveals that availability of equipment does not necessarily translate into execution of services. Mammograms examination capacity was estimated based on the number of functional mammogram machines using CNES. At the national level, estimated production capacity exceeded the number of tests needed by 153%. Nevertheless, when considering the actual utilization of these machines a different picture emerges. Even if functional mammogram machines exist, actual production is low across the country at 37% of capacity. This shows that there are other constraints to increasing their use. These may include the unavailability of qualified health workers (radiology technicians, radiologists, and breast clinics) and low maintenance of equipment/machines.
- There is also significant variation in the availability of cancer care hospitals and oncologists across regions in Brazil (Fig. 6a and b) where regions that are better off have more resources. This may explain why states/regions that are better off economically (e.g. the southeast) have, e.g. higher coverage rates of breast cancer screening as shown previously. According to data from the national registry of health institutions from 2013, Brazil has nearly 1.5 cancer hospital and 23 oncologists per million people. However, regional averages range from 0.6 hospitals per million in the north to 2.3 per million in the central west, and 7 oncologists per million in the north to 32 per million in the southeast. Similar disparities exist for other cancer care resources, such as radiation therapy units, mammography units, MRIs and CT scanners.

**Fig. 7:**
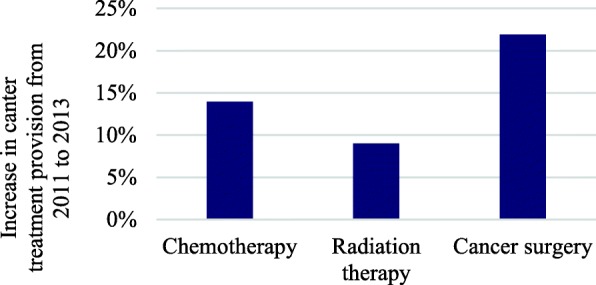
Percentage increase in cancer treatment provision between 2011 and 2013, by services

Data on government spending on cancer is limited. Existing data (the Federal Audit Authority estimated spending on cancer care to reach R$1.9 billion in 2010) only includes federal spending of hospital procedures linked to cancer and omits large amounts of resources spent by states and municipalities [21]. Since an important factor for determining the size of federal transfers to the state and municipal levels is reported volume of cancer related procedures, it is relevant to consider this indicator. Figure 7 shows cancer related procedures increased between 9 and 22% over a two-year period (2011–2013). While these estimates indicate that the cost for cancer care is increasing in Brazil, available data do not permit a more detailed analysis related to how resources are being spent on cancer and fairness in financing.

## Discussion

Through this empirical assessment on equity and the right to health along patient care pathways for breast and cervical cancer in Brazil, it is shown that patients’ right to health is systematically violated at different stages of the patient pathway.

In terms of *early detection and screening*, the analysis reveals that at the national level only 65% and 54% of the estimated need for mammography exams and Pap smear exams respectively is met. There is large variation across states in the extent to which screening needs for breast and cervical cancer are met, with a tendency for lower screening rates among the poorest groups of society. Also, coverage rates of breast cancer screenings are higher in states that are better off economically. Half of mammography screenings is outside the target population, which may be caused by implicit prioritization by health workers. This represents waste and could potentially be harmful for women who could get exposed to unnecessary radiation associated with mammography. Quality of cervical cancer diagnostics procedures is also a key concern: almost half (46%) of collected samples for cervical cancer screening is never completed, with consequences for patients, who face repeated tests and the stress that this implies. This is an important contributor to low screening rates for cervical cancer and raises concern about the training and knowledge of healthcare workers in this area, the coordination between primary care providers and diagnostics providers and the quality of laboratory services.

The patients’ right to *accurate diagnosis at an early stage* is also violated. The analysis confirms findings of previous research [28] and shows that few cases of breast cancer (26%) and cervical cancer (39%) are diagnosed at an early stage (stage 0 or I), thereby reducing the survival prospects of patients. There is large variation in performance among states, particularly for early diagnosis of cervical cancer, with a tendency of poorer states faring worse than wealthy states in terms of early diagnosis of the two cancers. Contributing factors to late diagnosis include: challenges with the quality of diagnostic exams for cervical cancer, low access to specialist care and more sensitive diagnostics procedures (e.g. biopsy and FNA) needed to confirm diagnosis and no effective prioritization of patients with advanced cancer in confirmation of diagnosis.

While the right to *prompt access to treatment* is now codified in the law that guarantees treatment within 60 days of confirmed diagnosis, the study shows that waiting times are not systematically collected and monitored along the patient pathway. Available data on the time between confirmed diagnosis and treatment suggests that waiting times are long and depend on patients’ health-seeking behaviors. Waiting times are significantly longer for patients that follow the recommended patient pathway and are diagnosed outside the hospital compared to these who seek care directly at the hospital level and receive both diagnosis and treatment in a hospital (76 vs. 29 days for breast cancer and 82 vs. 29 days for cervical cancer). For patients that go through the recommended patient pathway, i.e. diagnosis outside the hospital and referral to treatment in the hospital, most of states have difficulty meeting the 60-day waiting time guarantee stipulated in the law. While data is not available on the socio-economic status of patients for this variable, this finding may be a sign that patients who are less informed and weak may fare worse in a system where the recommended pathway does not deliver on timely cancer treatment. Consistent with the long waiting times and the advance stage at which many women receive confirmed diagnosis, a larger share of women starts treatment with chemotherapy (82.2%), the most common treatment when the cancer is detected late, compared to surgery (50.6%).

The paper shows how inequalities and rights violations are the result of complex interactions of different parts of the health system. At first glance, Brazil is relatively well resourced from an international perspective as far as oncologists per capita and availability of technology for effective cancer care are concerned. However, detailed analysis reveals that availability of equipment does not necessarily translate into execution of services as e.g. mammogram machines produce below capacity. The unequal distribution of oncologists and cancer hospitals across the country are likely contributing to difficulties and delays in physical access to cancer care observed in the study. An in-depth capacity utilization assessment could be useful to better understand existing capacity constraints and monitor the use of equipment, facilities and workforce dedicated to cancer care.

The paper also shows that important data points are not available, e.g., the financing of cancer care. This makes it difficult to draw firm conclusions on the health system determinants for delivery of cancer care.

Yet, this type of detailed health system analysis, not only exposes systemic inefficiencies along the patient pathway, it also sheds light on how these inefficiencies can be addressed to improve the right to health. For instance, several measures can be taken to reduce waiting times and provide prompter access to treatments. The coordination of care between the primary care level and the hospital level as well as units carrying out diagnostics procedures could be improved by establishing clear accountability frameworks and incentives for compliance with agreed rules, fast-track pathways for patients with advanced cancer could be introduced, and the use of existing oncologists and cancer care hospitals could be optimized and become more productive.

A key limitation of this study is the lack of data along many dimensions such as race, sexuality and in some cases socio-economic status. The analysis showed that when such analysis could be completed, e.g. for coverage of screening for breast and cervical cancer, SUS is not fair, nor pro-poor. Investments in better data and more systematic use of the same is therefore not just important to improving the management of the health system but also to assess health equity in a specific area or to monitoring rights violations. In the case of Brazil, a population-based national cancer registry is critical to better monitor the equity of cancer care. Patient-based surveys, with questions related to cancer care, could also be administered on a regular basis to determine how well the system serves its citizens. Improved information systems and use of data by HWs could enhance coordination of care and better data on e.g. waiting times and treatment choices, would allow for health system performance monitoring and national and international benchmarking.

Judicialization is potentially an important tool to ensure effective coverage of interventions and the maximization of equity in access to quality of care. With this paper we argue that detailed health system analysis along the patient pathway, with data that is disaggregated along axes of gender, race, socio-economic status, sexuality, and disability, can provide a powerful complimentary tool in research on the right to health. Health system research is valuable because it can pinpoint exactly how and where citizens’ right to health is violated in practice and provide strategies to address these issues. It can also be used to engage citizens and patient groups and give them voice and evidence to argue their case and thereby open doors for popular input to shape health policy and hold states and other parties accountable. Giving voice to vulnerable populations and enabling them to change their conditions of vulnerability are critical pieces of the human rights-based approach [34]. As pointed out by Yamin (2014), health systems are social institutions whose design and organization depends on political struggles, which ultimately determine what health outcomes they produce and how health and ill health are distributed across citizens in societies [8].

## Conclusions

The study reveals that there are large variations between states and patients, where the poorest states and patients fare worse on key indicators related to breast and cervical cancer care. More broadly, the paper shows the importance of collecting data both on patient characteristics and health system performance and carry out detailed health system analysis for exposing, empirically, rights violations and for identifying how they can be addressed.

## Additional file

Additional file 1: **Table S1**. Summary description of methodological approaches used in the qualitative study in Bahia (DOCX 17 kb)

## Abbreviations

CNES: Cadastro Nacional de Estabelecimentos de Saúde, Brazilian Registry of Health Facilities
CT: Computerized Tomography
FNA: Fine-Needle Aspiration
HCR: Registro Hospital de Câncer, Hospital Cancer Registry
HDI: Human Development Index
HPV: Human papillomavirus
HW: Health Worker
INCA: Instituto Nacional de Câncer, The Brazilian National Cancer Institute
MOH: Ministério da Saúde, Ministry of Health
MRI: Magnetic Resonance Imaging
OECD: Organization for Economic Co-operation and Development
PNAD: Pesquisa Nacional por Amostra de Domicílios, The Brazilian Institute of Geography and Statistics' yearly household survey
SESAB: Secretaria de Saúde do Estado da Bahia, State Health Secretariat of Bahia
SIA: Outpatient Information System
SISCAN: Sistema de Informação do Câncer, Cancer Information System
SISCOLO: Sistema de Informação do Câncer de Colo Uterino, Cervical Cancer Control Program Information System
SISMAMA: Sistema de Informação do Câncer de Mama, Breast Cancer Control Program Information System
SUS: Sistema Único de Saúde, Unified Health System
TCU: Tribunal de Contas da União, Court of Audits
WHO: World Health Organization

## Footnotes

1 Brazil has made significant progress in reducing inequality, the most commonly used inequality measure – the Gini coefficient (the closer to 1, the more unequal) – declined from 0.60 in 1990 to 0.51 in 2014 [35], but regional inequalities remain. For instance, moderate poverty was 15.1% and 17.6% in the north and northeast regions, while the same number for the south and southeast regions was 3.3% and 4.4%, respectively [36]

2 Biehl et al. [37] challenge this research based on their analysis of 1262 lawsuits from Rio Grande do Sul and find that most patient-litigants are poor and older individuals. They conclude that there are regional differences that should not be fit into one singular narrative.

3 Existing cancer registries in Brazil record data from specific capital cities, which is then used to estimate incidence and mortality rates across a larger sample of the population. However, this evidence presents a narrow perspective of select population-based cancer registries within a small segment of the population living in areas of Brazil with higher economic status. This data is not nationally representative and therefore problematic for cross-country comparisons. The authors explored using the data. However, the quality of the data was poor. There were large discrepancies among the capitals in terms of reporting information. Some of the registries had not been fed for years. Furthermore, for the registries with more recent data, data management was sometimes outsourced to third parties and without standardized guidelines for managing the data this led to inconsistencies in data reporting. For all these reasons, the authors decided to not use the data in this study.

4 MOH Ordinance on cancer treatment waiting time guarantee (Portaria No 876 de 16/5/2014).

## References

[1] Yamin AE (2017). Taking the right to Health seriously: implications for Health systems, courts, and achieving universal Health coverage. Hum Rights Q.

[2] WHO. Making fair choices on the path to universal health coverage: Final report of the WHO consultative group on equity and universal Health coverage. Geneva: World Health Organization. 2014. http://apps.who.int/iris/bitstream/10665/112671/1/9789241507158_eng.pdf?ua=1. Accessed 30 Apr 2018.

[3] WHO. Positioning health in the post-2015 development agenda. WHO discussion paper. October 2012. http://www.who.int/topics/millennium_development_goals/post2015/WHOdiscussionpaper_October2012.pdf. Accessed 30 Apr 2018.

[4] WHO. Constitution of the World Health Organization as adopted by the International Health Conference, New York 19–22 June, 1946; signed on 22 July 1946 by the representatives of 61 States (Official Records of the World Health Organization no 2).

[5] Braveman P, Gruskin S (2003). Defining equity in health. J Epidemiol Community Health.

[6] WHO. Health System: Equity. http://www.who.int/healthsystems/topics/equity/en/. Accessed 30 Apr 2018.

[7] Chieffi AL, De Cassia Barata Barradas R, Golbaum M (2017). Legal access to medications: a threat to Brazil’s public health system?. BMC Health Serv Res.

[8] Yamin AE (2014). Editorial: Promoting equity in health: what role for courts?. Health Hum Rights..

[9] Gragnolati M, Lindelow M , Couttolenc B. Twenty years of health system reform in Brazil: an assessment of the Sistema Unico de Saude. World Bank Publications 2013.

[10] Barreto ML, Rasella D, Machado DB, Aquino R, Lima D, Garcia LP, et al. Monitoring and evaluating Progress towards universal Health coverage in Brazil. PLoS Med. 2014;11(9). 10.1371/journal.pmed.1001692 PMC4171375 25243676

[11] Hone T, Rasella D, Barreto M (2017). Large reductions in amenable mortality associated with brazil’s primary care expansion and strong health governance. Health Aff.

[12] Massuda A, Hone T, Leles FAG (2018). The Brazilian health system at crossroads: progress, crisis and resilience. BMJ Global Health.

[13] Duarte C S And Bergamo Braga, PV. The utilization of clinical protocols and therapeutic guidelines and the rationalization of judicialization for the right to health. R. Dir. sanit., São Paulo; 2017;18:171–190. file:///C:/Users/wb304103/Downloads/wp17225%20(2).pdf.

[14] Ferraz OLM, Gloppen S, Maestad O, Rakner L, Sinding Aasen H, Gloppen S, Magnussen A-M, Nilsen (2014). Judging the price of life: cost consideration in right-to-health litigation. Juridification and social citizenship in the welfare state.

[15] Ferraz OLM, Yamin A, Gloppen S (2011). Health Inequalities, Rights and Courts: The Social Impact of the Judicialization of Health in Brazil. Litigating the Right to Health: Can courts bring more justice to health systems?.

[16] Chieffi AL, Barradas Barata R (2009). Judicialização Da Política Pública de Assistência Farmacêutica E Eqüidade. Cadernos de Saúde Pública.

[17] UN Committee on Economic, Social and Cultural Rights. General Comment no. 14: The right to the Highest Attainable Standard of Health (Art. 12. paragraph 8).

[18] Ministério de Saúde (Brasil). (2014). Indicadores e Dados Básicos - Brasil - 2012. Brasilia. http://tabnet.datasus.gov.br/cgi/idb2012/matriz.htm. Accessed 19 Feb 2019.

[19] Brasil Ministério da Saúde. Instituto Nacional do Câncer (INCA). 2018. http://www1.inca.gov.br/rbc/n_64/v01/pdf/15-review-estimate-2018-cancer-incidence-in-brazil.pdf. Accessed 30 Apr 2018.

[20] United Nations, Department of Economic and Social Affairs, Population Division. World population prospects: the 2015 revision, key findings and advance tables. Working paper no. ESA/P/WP.241. New York: United Nations; 2015. file:///C:/Users/wb304103/Downloads/wp17225%20(2).pdf.

[21] Ferraz OLM. 2013. The right to Health in the courts of Brazil: worsening Health inequities?. Health and Human Rights Journal 2013;08:29. 20845840

[22] Luciane Cruz L, Barberato-Filho S, Chad Costa A, Garcia Serpa Osorio-de-Castro C (2010). Uso Racional de Medicamentos Antineoplásicos E Ações Judiciais No Estado de São Paulo. Rev Saude Publica.

[23] Fabiola Sulpino V, Zucchi P (2007). Distortions to national drug policy caused by lawsuits in Brazil. Rev Saude Publica.

[24] Rezende M, Hilton CR, Koch A, de Almeida Figueiredo J, Santos Thuler LC (2009). Causas Do Retardo Na Confirmação Diagnóstica de Lesões Mamárias Em Mulheres Atendidas Em Um Centro de Referência Do Sistema Único de Saúde No Rio de Janeiro. Revista Brasileira de Ginecologia E Obstetrícia.

[25] Barros AF, Uemura G, Lessa Soares de Macedo J (2013). Tempo Para Acesso Ao Tratamento Do Câncer de Mama No Distrito Federal, Brasil Central. Revista Brasileira de Ginecologia E Obstetrícia..

[26] Freitas-Junior R, Reis Gonzaga CM, Aires Freitas NM, Martins E, de Cassia de Maio Dardes R (2012). Disparities in female breast Cancer mortality rates in Brazil between 1980 and 2009. Hospital das Clínicas da Faculdade de Medicina da Universidade de São Paulo.

[27] Simon, S., Bines J., Barrios C. Clinical characteristics and outcome of treatment of Brazilian women with breast cancer treated at public and private institutions—the AMAZONE project of the Brazilian breast Cancer study group (GBECAM). 32nd annual CTRC-AACR san Antonio breast Cancer symposium, USA; Dec 10–13, 2009.

[28] Tribunal de Contas Da União (TCU) (2011). Relatório de Auditoria Operacional: Política Nacional de Atenção Oncológica.

[29] ANS. Agencia Nacional de Saude Suplementar. General Statistics. http://www.ans.gov.br/perfil-do-setor/dados-gerais. Accessed 11 Aug 2018.

[30] OECD. Cancer Care*:* Assuring quality to improve survival, OECD Health Policy Studies 2013. 10.1787/9789264181052-en Accessed 30 Apr 2018.

[31] WHO. Cancer: Cancer Prevention. http://www.who.int/cancer/prevention/en/. Accessed 30 Apr 2018.

[32] Brasil Ministério da Saúde. 2015. Sistemas de Informação do Programa Nacional de Imunizações (SI-PNI). Estratégia de Vacinação contra HPV - Coberturas vacinais - HPV Quadrivalente D2 - Sexo feminino de 9 a 14 anos de idade Total Brasil – 2014.

[33] WHO. WHO Position Paper on Mammography Screening. http://apps.who.int/iris/bitstream/handle/10665/137339/9789241507936_eng.pdf;jsessionid=335F626666797563C2F09E15580E09C4?sequence=1. Accessed 30 Apr 2018.

[34] London L (2008). What is a human-rights based approach to health and does it matter?. Health Human Rights J.

[35] Góes C., and Karpowicz, I. Inequality in Brazil: a regional perspective. IMF working paper no. 17/225. Washington: International Monetary Fund.

[36] World Bank. 2016 Brazil - systematic country diagnostic (English). Washington, D.C. : world bank group. http://documents.worldbank.org/curated/en/180351467995438283/Brazil-Systematic-country-diagnostic. Accessed 14 Jan 2019.

[37] Biehl J., Socal MP., and Amon JJ. The Judicialization of Health and the quest for state accountability: evidence from 1,262 lawsuits for access to medicines in southern Brazil. Health Hum Rights 2016; 06:18(1):209–220. PMC5070692 27781011

