# Supplementary material for: Realizing the right to health in Brazil’s Unified Health System through the lens of breast and cervical cancer
Source: Int J Equity Health. 2019 Jun 3;18:39. doi: 10.1186/s12939-019-0938-x (PMC6545675; doi:10.1186/s12939-019-0938-x)
Supplement: Supplementary file 1 — Table S1. Summary description of methodological approaches used in the qualitative study in Bahia (DOCX 17 kb) [file 12939_2019_938_MOESM1_ESM.docx]

Additional file 1: Table S1. Summary description of methodological approaches used in the qualitative study in Bahia

| **Methodology** | **Objectives** | **Selection criteria** | **Sample** | **Approach** | **Observations** |
| --- | --- | --- | --- | --- | --- |
| **Roundtable discussions with health workers (HW)** | -Understand the organization of cancer care.  -Identify challenges perceived by HW | -HW in the east-macro region working under the responsibility of the local health department.  3 municipalities/ health region  -At least one HW per area:  1) Primary care (with focus on doctors, nurses, nursing technicians, and community health agent)  2) Specialized care  3) Regulation and control  4) Information systems | Total of 116 participants in 12 roundtables (3 per health region) Geographical areas (by health region): Salvador: Salvador, Saubara e Vera Cruz Camaçari: Camaçari, Pojuca e Dias d’Ávila Santo Antônio de Jesus: Santo Antônio de Jesus, Amargosa, Itatim  Cruz das Almas: Cruz das Almas, Governador Mangabeira e Maragogipe | Roundtable 1:  Introduction, contextualization of study, agreement of the terms of participation  Roundtable 2: Presentation and discussion of two clinical cases  Roundtable 3: Presentation of first clinical case and evaluation of roundtable. | Participation decreased in second and third roundtable in some regions.  The municipality manger participated in some roundtables this may have influenced participation. |
| **Focus group discussions** | -Deepen the understanding of organization of care and patient pathways.  -Identify weaknesses in system design. | -Representatives from the State Health Secretariat of Bahia (SESAB) and the municipality of Salvador, as well as HWs working in cancer care.  -Individual were identified by SESAB and the municipality of Salvador, and invitations were sent by email. | Total of 17 participants from SESAB, the municipality of Salvador, primary care and the State Center for Oncology. | Structured interviews based on questionnaires focused on access, regulation & contracting, primary and specialized care  Focus group 1: 11 participants  Follow-up meeting to validate findings: 6 participants | Some participants dropped out from the primary care level and the State Center of Oncology. This resulted in primary focus on medium and high complexity care. |
| **Direct observations and semi-structured interviews** | -Construct hypothetical patient pathway.  -Identify how to get access to screening tests, confirmed diagnosis, specialist appointments. | 3 sites were selected based on the level of care they represented (see description of sample). | Itaim municipality (217 km from Salvador) to study referrals from primary care (rural).  Camacari health region (54 km from Salvador) with 5 municipalities (Conde, Dias d'Ávila, Mata de Sao Joao, Pojuca and Simões Filho) to study referrals from primary care (urban) and medium complexity care. A specialized Oncology Center [CEONC] was visited.  City of Salvador: to study high complexity care. The State Center for Oncology and Aristides Hospital Maltez were visited. | Structured interviews on-site to construct hypothetical patient pathway based on questionnaire as well as direct observations in waiting rooms etc. | Because of time limitations it was not possible to visit a diagnostic center in Salvador as well as teams that support home-based cancer patients. . |
| **Review of policies and program** | Identify relevant policies and programs that impact on organization of cares in the state. | N/A | State of Bahia. | Representatives from SESAB responded to a questionnaire, which was adapted from a conceptual model developed by the OECD to assess cancer care.  Consultant collected information on policies and programs at state level. | Respondent did not reply to all questions. |
